# History and Outcome of Febrile Neutropenia Outside the Oncology Setting: A Retrospective Study of 76 Cases Related to Non-Chemotherapy Drugs

**DOI:** 10.3390/jcm6100092

**Published:** 2017-09-26

**Authors:** Emmanuel Andrès, Rachel Mourot-Cottet, Frédéric Maloisel, Olivier Keller, Thomas Vogel, François Séverac, Martine Tebacher, Jacques-Eric Gottenberg, Jean-Christophe Weber, Georges Kaltenbach, Bernard Goichot, Jean Sibilia, Anne-Sophie Korganow, Raoul Herbrecht

**Affiliations:** 1 Departments of Internal, Strasbourg University Hospitals, Strasbourg 67000, France; rachel.mourotcottet@chru-strasbourg.fr (R.M.-C.); olivier.keller@chru-strasbourg.fr (O.K.); jean-christophe.weber@chru-strasbourg.fr (J.-C.W.); bernard.goichot@chru-strasbourg.fr (B.G.); anne-sophie.korganow@chru-strasbourg.fr (A.-S.K.); 2 Departments of Onco-hematology, Strasbourg University Hospitals, Strasbourg 67000, France; f.maloisel@solcrr.org (F.M.); raoul.herbrecht@evc.net (R.H.); 3 Departments of Geriatrics, Strasbourg University Hospitals, Strasbourg 67000, France; thomas.vogel@chru-strasbourg.fr (T.V.); georges.kaltenbach@chru-strasbourg.fr (G.K.); 4 Departments of Statistics, Strasbourg University Hospitals, Strasbourg 67000, France; francois.severac@chru-strasbourg.fr; 5 Regional Pharmacovigilance Centre of Alsace, Strasbourg 67000, France; martine.tebacher@chru-strasbourg.fr; 6 Departments of Rheumatology, Strasbourg University Hospitals, Strasbourg 67000, France; jacques-eric.gottenberg@chru-strasbourg.fr (J.-E.G.); jean.sibilia@chru-strasbourg.fr (J.S.)

**Keywords:** fever, neutropenia, agranulocytosis, drug, infection, hematopoietic growth factor

## Abstract

Background: Despite major advances in its prevention and treatment, febrile neutropenia remains a most concerning complication of cancer chemotherapy. Outside the oncology setting, however, only few data are currently available on febrile neutropenia related to non-chemotherapy drugs. We report here data on 76 patients with febrile neutropenia related to non-chemotherapy drugs, followed up in a referral center within a university hospital. Patients and methods: Data from 76 patients with idiosyncratic drug-induced febrile neutropenia were retrospectively reviewed. All cases were extracted from a cohort study on agranulocytosis conducted at the Strasbourg University Hospital (Strasbourg, France). Results: Mean patient age was 52.2 years old (range: 18–93) and gender ratio (F/M) 1.6, with several comorbidities present in 86.8% of patients. The most common causative drugs were: antibiotics (37.4%), antithyroid drugs (17.2%), neuroleptic and anti-epileptic agents (13.1%), non-steroidal anti-inflammatory agents and analgesics (8%), and platelet aggregation inhibitors (8%). Main clinical presentations upon hospitalization included isolated fever (30%), sore throat, acute tonsillitis and sinusitis (18.4%), documented pneumonia (18.4%), septicemia (14.5%), and septic shock (6.6%). Mean neutrophil count at nadir was 0.13 × 10(9)/L (range: 0–0.48). While in hospital, 22 patients (28.9%) worsened clinically and required intensive care unit placement. All patients were promptly treated with broad-spectrum antibiotics, and 45 (59.2%) with hematopoietic growth factors. Mean duration of hematological recovery (neutrophil count ≥1.5 × 10(9)/L) was 7.5 days (range: 2–21), which was reduced to 0.7 days (range: 2–16) (*p* = 0.089) with hematopoietic growth factors. Outcome was favorable in 89.5% of patients, whereas eight died. Conclusions: Like in oncology and myelosuppressive chemotherapy settings, idiosyncratic febrile neutropenia is typically serious, about 40% of patients exhibiting severe pneumonia, septicemia, and septic shock, with a mortality rate of 10%. Like in febrile, chemotherapy-related neutropenia, modern and timely management (immediate broad spectrum antibiotherapy, hematopoietic growth factors) may reduce infection-related mortality. All practitioners should be aware of this potential side-effect that may even occur in the event of “daily medication” exposure.

## 1. Introduction

Despite major advances in its prevention and treatment, febrile neutropenia remains a most concerning complication of cancer chemotherapy, especially in patients receiving myelosuppressive drugs [1]. Chemotherapy-induced febrile neutropenia is traditionally managed via hospital admission for parenteral antibiotics until neutropenia resolves [2]. Recent studies have explored risk stratification, along with the safety of managing “low-risk” patients as outpatients [3]. In this cancer context, recent studies documented the usefulness of granulocyte colony-stimulating factor and its pegylated forms for prophylaxis in myelosuppressive chemotherapy or for patients with a first episode of febrile neutropenia [4].

Outside the chemotherapy setting, only few data are currently available in regards to febrile neutropenia, especially whilst related to non-chemotherapy drugs, called “idiosyncratic agranulocytosis” [5,6]. In addition, only few data dealing with congenital neutropenia have so far been published [7].

In the present paper, we report on 76 patients with established febrile neutropenia related to non-chemotherapy drugs, followed up in a referral center within a university hospital.

## 2. Patients and Methods

### 2.1. Patientselection

All severe neutropenia cases related to non-chemotherapy drugs that have been reported in patients hospitalized in the Strasbourg University Hospitals (Strasbourg, France, a tertiary referral center) since the 1980’s were identified and included into a register (partial data published in [8]).

Patients were recruited from the Internal Medicine, Onco-Hematology, Geriatric Medicine, Rheumatology, and Digestive Surgery departments. Since our first publication on the topic of idiosyncratic drug-induced agranulocytosis [9], a protocol has been set up in our hospital to help manage these patients optimally (for details see the reference [6]).

### 2.2. Inclusion Criteria

Severe neutropenia or agranulocytosis was defined as absolute neutrophil count <0.5 × 10(9)/L. The inclusion and exclusion criteria have been listed in Table 1, with all cases fulfilling the international criteria of idiosyncratic drug-induced agranulocytosis [6,9]. Additionally, all cases met the criteria “*Vraisemblable*” (likely causative) or “*Très vraisemblable*” (very likely causative) from the French causality assessment method of adverse drug reactions [10]. All cases were reported to the Regional Pharmacovigilance Centre of Alsace, France.

In this study, we have only included febrile patients, defined as corporeal temperature >38.5 °C when neutropenia was detected, in accordance with the febrile neutropenia definition in the oncology setting [2]. 

### 2.3. Objective, Method, and Collected Data

The first study objective was to fully describe the clinical picture and outcome of patients with established febrile neutropenia related to non-chemotherapy drugs, at the time of discovery. All data were retrieved from patient files.

For each case, the patient file was consulted and assessed by two members of the monitoring committee, with each of the following factors recorded (when available): age; gender; medical history and comorbidities (such as diabetes mellitus, cardio- or respiratory disorders, renal failure, or systemic inflammatory disorders); clinical picture at diagnosis and disease progression; drugs administered (dose, route, start date, and withdrawal date); absolute white blood cell, hemoglobin, and platelet counts; bone marrow analysis; clinical features; time to reach a neutrophil count exceeding 1.5 × 10(9)/L (hematological recovery); use of hematopoietic growth factors (HGF), e.g., granulocyte-colony stimulating factor (G-CSF); number of days in hospital; final outcome, recourse to intensive care placement and mortality rate. 

### 2.4. Statistical Analysis

Data were expressed as mean and standard deviation (SD) and analyzed using the Mann-Whitney test and Student’s *t*-test for paired data. The distribution of quantitative variables was assessed graphically and using the Shapiro-Wilk test. Between-group comparison of quantitative variables was performed using the nonparametric Mann-Whitney test. The qualitative variables, presented as numbers and percentages, were analyzed using Pearson’s chi-squared or Fisher’s exact test, depending on patient numbers. Univariate analysis was carried out to assess the factors associated with death and/or intensive care unit admission. A *p*-value < 0.05 was considered statistically significant. Analyses were performed using the R Software (Version 3.2.2, R Development Core Team, Strasbourg, France).

### 2.5. Administrative Data

All data were registered into the French national database of drug side-effects (Réseau des Centres Régionaux de Pharmacovigilence (CRP)).

The data collected were subject to the French national data protection act (Commission Nationale Informatique et Liberté) (CNIL). This study received approval from the local ethics committee.

## 3. Results

From January 1984 to January 2014 (30 years), 76 patients with febrile neutropenia related to non-chemotherapy drugs, at time of the discovery, were registered. At the time of detecting this drug-related adverse effect, eight patients (10.5%) were already hospitalized for another reason (“in patients”); the other patients were treated at home (“out patients”).

### 3.1. Patient Baseline Characteristics

All patients were Caucasian (*n* = 76). Mean and median age were 52.2 and 56 years (range: 18–93), respectively, with 16 patients (21.1%) younger than 50 years, and 51 (67.1%) younger than 75 years. Six patients were aged 85 years or more (7.9%). The female/male ratio was 1.6.

Several comorbidities were found in 86.8% of cases (*n* = 66), consisting primarily of: thyroid disorders (mainly Grave’s disease) (*n* = 17, 22.4%); arterial hypertension (*n* = 13, 17.1%); cardiac disorders (cardiac failure, atrial fibrillation, myocardial infarction, arteritis of lower limbs, or stroke) (*n* = 13, 17.1%); chronic renal failure (creatinin clearance <60 mL/min) (*n* = 13, 17.1%); neuro-psychiatric disorders (psychosis, schizophrenia, dementia, epilepsy, anorexia, and depression) (*n* = 11, 14.5%). 

### 3.2. Causative Drugs

A single drug was documented as “causative” or “likely causative” in all except 12 cases (15.8%), for which two to four drugs were suspected. The causative drugs have been listed in Table 2. The respective causative drugs were stopped within the first 48 h of admission in 123 patients (60.6%). 

The main drug families found to be causative were: antibiotics (*n* = 37, 37.4%), especially β-lactams (*n* = 21) and cotrimoxazole (*n* = 6); antithyroid drugs (*n* = 17, 17.2%); neuroleptic and anti-epileptic agents (*n* = 13, 13.1%); non-steroidal anti-inflammatory agents and analgesics (*n* = 8, 8%); platelet aggregation inhibitors (*n* = 8, 8%), especially ticlopidine (*n* = 5) (Table 2). Since 1990 and 2000, no case of noramidopyrine- and ticlopidine-induced agranulocytosis has been observed, respectively. Conversely, cases of febrile neutropenia associated with either clozapine or antiviral agents have been observed only since 2000 and 2005, respectively. With the exception of four, all cases of antibiotic-induced neutropenia were observed in hospitalized patients. 

In half of cases (*n* = 40), patients were treated with at least three drugs (mean number of drugs: 5.2; range: 1–13). Overall, 21 patients (27.6%) did not take another drug in addition to the causative drug within the 10 days preceding febrile neutropenia detection. There was only one case of self-medication. The mean and median durations of the suspected drug intake were 54 and 30 days (range: 3–120), respectively. A quarter of the patients received the causative drug for at least 30 days.

### 3.3. Clinical Manifestations

The main discovery circumstance, based on the inclusion criteria used (febrile neutropenia), was fever occurring as isolated fever (of unknown origin) in 70 patients (92.1%). Six patients (7.9%) exhibited fever related to pre-existing pneumonia (*n* = 2), pelvic and abdominal abscess (*n* = 2), and meningitis (*n* = 2).

The main clinical presentations upon hospitalization were as follows: isolated fever (*n* = 23, 30%); sore throat, acute tonsillitis and sinusitis (*n* = 14, 18.4%), with one case of maxillary sinus mucormysosis; documented pneumonia (*n* = 14, 18.4%), with one case of pulmonary aspergillosis and one case of acute respiratory distress syndrome; septicemia (*n* = 11, 14.5%); septic shock (*n* = 5, 6.6%). Table 3 summarizes data of the remaining symptomatic patients with documented infection (*n* = 9, 11.8%). 

Microbiological documentation was obtained from 21 patients (31.8%) (data available for *n* = 66), with mainly Gram-negative bacilli (*n* = 8) and *Staphylococcus* sp. (*n* = 7) (Table 4). 

While in hospital, 22 patients (28.9%) worsened clinically and exhibited features of severe sepsis (*n* = 3), septic shock (*n* = 10), or systemic inflammatory response syndrome (SIRS) (*n* = 9).

### 3.4. Hematological Data

At diagnosis, the mean and median neutrophil counts were 0.13 and 0.06 × 10(9)/L (range: 0–0.48), respectively. In total, 59.2% of patients (*n* = 45) had neutrophil levels <0.1 × 10(9)/L. At the neutrophil decrease nadir, the mean and median neutrophil counts were 0.06 and 0 × 10(9)/L (range: 0–0.4). At nadir, a total of 93.4% of the patients (*n* = 71) displayed neutrophil levels <0.1 × 10(9)/L.

Overall, 28 patients (36.8%) exhibited isolated neutropenia, without any modification in red blood cells or platelets. The mean and median hemoglobin levels were 112.1 and 110 g/L (range: 74–153), respectively, while 50 patients (65.8%) suffered from anemia (hemoglobin <120 g/L). The mean and median platelet counts were 217 and 203 × 10(9)/L (range: 7–694), while 23 patients (11.3%) had thrombocytopenia (platelet count <100 × 10(9)/L). 

Bone-marrow analysis (data available for *n* = 46) primarily detected myeloid hypocellularity with apparent cessation of myeloid precursor maturation (at the promyeloid stage) in 73.9% (*n* = 34).

### 3.5. Duration of Hematological Recovery and Response to Hematopoietic Growth Factors

The mean and median durations of hematological recovery (from initial neutropenia documentation to neutrophil count ≥1.5 × 10(9)/L) were 7.5 and 6 days (range: 2–21), respectively. The mean and median durations for achieving neutrophil counts ≥0.5 × 10(9)/L were 6.9 and 5 days (range: 1–21). 

Granulocyte-colony stimulating factor (G-CSF) (administered subcutaneously at a fixed dose of 300 μg/day) was given to 45 patients (59.2%), particularly those with neutrophil counts <0.1 × 10(9)/L, severe clinical infectious features (e.g., collapse, septicemia, or extensive pneumonia), or renal failure. This hemopoetic growth factor (HGF) was administered for mean and median durations of 6.1 and 5 days (range: 1–18), respectively. For these 45 patients, the mean duration of hematological recovery was reduced to 0.7 days (range: 2–16), from 7.5 to 6.9 days (*p* = 0.089). The mean durations of antibiotherapy and hospitalization were not impacted when using HGF and consisted of 14.7 (range: 7–35) and 23.7 days (range: 5–74), respectively (all *p* > 0.4) (data not developed).

### 3.6. Management, Duration of Hospitalization and Outcome

All patients were hospitalized and treated immediately (within the first 24 h) with broad-spectrum parenteral antibiotherapy, mainly piperacilline (12 g/day) or cefotaxime (3 g/day), associated with netromycine (5 mg/Kg/day) or amikacine (15 mg/Kg/day) in sepsis cases, except in the event of β-lactam allergy or β-lactam-induced agranulocytosis. In a second step, the antimicrobial therapy was adapted according to microbes and antibiogram results, comprising mainly glycopeptide antibiotics and penems, associated with amphotericin or voriconazole in fungal infection cases. The mean and median durations of antibiotherapy were 18.3 and 15 days (range: 7–120), respectively. Forty-five patients (59.2%) were treated with HGF.

The mean and median hospitalization durations (available for 50 patients) were 26.3 and 12 days (range: 5–200), respectively. Eleven patients (21.6%; data available for 51 patients) required intensive care placement. In univariate analysis, there was no risk factor for admission to intensive care unit identified (*p* for all data not significant; data not developed).

Outcome was favorable in 89.5% of subjects (*n* = 68), whereas eight patients (10.5%) died of either uncontrolled septic shock due to *Staphylococcus aureus* and *Pseudomonas aeruginosa* (*n* = 5) or aggravation of deep abdominal and pelvic abscess (*n* = 2) (Table 5). The remaining patient died of hemorrhagic stroke (no relation with the febrile neutropenia). In univariate analysis, there was no risk factor for death identified (*p* for all data not significant; data not developed). Four of these eight patients who died were treated with HGF. 

## 4. Discussion

To our knowledge, this is one of the first studies focused on febrile neutropenia outside the oncology setting, with a consequent number of patients from a single center entered, with well-documented febrile neutropenia related to non-chemotherapy drugs (as “daily medication”) and managed using the same procedure. To our knowledge, two others studies have recently been published in such setting, one in non-immunocompromised children who attended an emergency department for febrile neutropenia [11], and the second one in adult and child patients with febrile neutropenia [12]. Our study’s novelty is that it is the first to investigate febrile neutropenia defined as established and documented idiosyncratic non-chemotherapy neutropenia.

Our patient diagnoses corresponded to both definitions of febrile neutropenia [2] and idiosyncratic drug-induced agranulocytosis [5,6] (Table 1). All patients exhibited unquestionable febrile neutropenia outside the oncology context: mean neutrophil counts of 0.06 × 10(9)/L (range: 0–0.4) at the neutrophil decrease nadir; 93.4% of patients with neutrophil levels <0.1 × 10(9)/L at nadir. In all cases, the principal diagnostic criterion for idiosyncratic agranulocytosis, namely complete hematological recovery following causative drug removal [9], was fulfilled (except for the five patients who died of uncontrolled septic shock). As agranulocytosis is a life-threatening condition, no patient was re-challenged with the incriminated drug (“theoretical method of reference”).

In terms of the severity of infectious manifestations, clinical features observed in our population (Table 3) did not differ from those observed in other idiosyncratic agranulocytosis series, involving hospitalized patients of all ages, [5,13]. In our population, the main clinical pictures upon hospitalization were pneumonia (18.4%), septicemia (14.5%), and septic shock (6.6%). Only one-third of patients exhibited isolated fever. In the course of hospitalization, 28.9% of our patients displayed features of severe sepsis, septic shock, and/or SIRS, whereas 21.6% required intensive care placement. As discussed elsewhere, the severity of the clinical manifestations we have reported from our series were probably accounted for by neutropenia severity (mean neutrophil counts of 0.06 × 10(9)/L) and perhaps by the patient types we had selected (patient referred to a referral center).

These reported clinical features differ from those observed in febrile neutropenia series involving chemotherapy-treated patients, particularly with myelosuppressive chemotherapy [1,2]. In this context, our patients did not exhibit deep invasive mycosis (except in one case of *Aspergillus* sp. pneumonia and another case of maxillary sinus mucormycosis), as often reported in oncology and haematology patients [14,15]. Another observation is that our patients did not suffer from the following conditions: mucositis related to radio- or chemotherapy; deep immune deficiency related to malignant disorders associated to the neutropenia; exclusive “central” neutropenia (in opposition of “peripheral” neutropenia) related to partial bone marrow abolition (26.1% of our patients) [1,2]. It must also be mentioned that in most cases, a brutal installation of neutropenia was observed, which was of relatively short duration. 

In our study, we observed a mortality rate of 10.5%. Of the eight death cases, only five elderly patients died of sepsis in relation to agranulocytosis, namely uncontrolled septic shock due to *Staphylococcus aureus* and *Pseudomonas aeruginosa*. The mortality in our population did not differ from the figures of other recent idiosyncratic drug-induced agranulocytosis series reporting rates from 5.4 to 11% [5,13]. This global mortality represents the upper bound of the mortality rates, despite our patients being relatively young (mean age: 52.2 years) and our team, as referral center, having wide experience of managing agranulocytosis. Explanations for these discrepancies may be the severity of the infectious pictures we encountered, in addition to that 86% of patients presented underlying diseases that were often not stabilized, especially cardiac, neuro-psychiatric, and renal disorders. In the Julia et al. study, renal failure was considered a poor prognostic indicator reflecting severe infections, in association with a neutrophil count <0.1 × 10(9)/L [13]. In addition, Maloisel et al. have demonstrated that in the event of severe neutropenia (absolute neutrophil count <0.1 × 10(9)/L), the prognosis of idiosyncratic agranulocytosis to be impacted by the following factors: age (75 years-old), infection severity (septicemia and septic shock), and comorbidities (particularly renal failure) [16].

This mortality rate slightly differs from figures published on chemotherapy-related febrile neutropenia series [1,2]. In oncology or hematology settings, febrile neutropenia has been shown responsible for considerable morbidity, given that 20–30% of patients exhibited complications requiring in-hospital management, with an overall in-hospital mortality of about 10% [1,2]. In this context, the mortality figures reported appear directly impacted by the prognosis of the underlying cancer. There is a clear relationship between the severity of neutropenia (neutrophil count <0.5 × 10(9)/L) and intensity of chemotherapy. In terms of risk for febrile neutropenia, the different therapeutic regimens have been classified classified as high-risk (>20%), intermediate-risk (10–20%), or low-risk (<10%) [1,2]. Thus, mortality was shown to vary according to the Multinational Association of Supportive Care in Cancer (MASCC) prognostic index: lower than 5% if the MASCC score was ≥21, but possibly as high as 40% if the MASCC score was <15 [2]. Other factors with a similar role are: old age, several comorbidities, and performance status (OMS or Charlson score) [1,17]. To our knowledge, these factors (dependent of host) have not been well-studied in the context of idiosyncratic agranulocytosis that is unrelated to chemotherapy, particularly febrile neutropenia cases.

The above-mentioned considerations should be instrumental in deciding whether a chemotherapy-treated patient should receive primary or secondary HGF prophylaxis to decrease the potential febrile neutropenia risk [1,4]. In our study, we were unable to identify a risk factor for death. HGF use (in curative perspective) did not appear to alter either patient disease progression or mortality. So far, no data are available for HGF prophylaxis, except for the few published case-reports of clozapine-induced agranulocytosis [18].

Outcome was favorable in 89.5% of cases. In our center, all patients underwent established care procedures (for details, see [6,8]) (Table 6). This protocol has been modeled based on that pertaining to febrile neutropenia management in the oncology setting [1]. In our opinion, this may account for our good results despite the severity of patient clinical manifestations. In our experience, appropriate management of septic complications of idiosyncratic agranulocytosis, using both broad-spectrum antibiotherapy and HGF, may improve the condition’s prognosis [6,16].

A faster hematological non-significant recovery (neutrophil count >1.5 × 10^9^/L) was observed in the HGF group: −0.7 days (from 7.5 to 6.9 days) (*p* = 0.089). Nevertheless, there were no other improvements observed in these patients in relation with HGF therapy, regarding antibiotherapy and hospitalization duration. This was also the case in the oncology setting. No impact was documented in the event of HGF administration for neutropenic febrile patient (“curative” use of HGF) [4]. While these results do not seem to be in line with those previously reported by our research team [6], they are consistent with those originating from larger HGF studies involving adult idiosyncratic drug-induced agranulocytosis patients [19,20]. For a number of hematologists, HGF usefulness in this setting is still a matter of debate. In line with this debate, the only available prospective randomized study (based on 24 patients with antithyroid-related IDIA) did not confirm the benefits of administering G-CSF [21]. It should, however, be mentioned that this negative study result may be accounted for by inappropriate G-CSF doses (100–200 μg/day) [6]. 

Of note is also that all our patients with febrile neutropenia were promptly hospitalized. For us, it appears mandatory that this consensual recommendation be an integral part of idiosyncratic agranulocytosis management [5,6]. This should, however, not be the case for chemotherapy-induced febrile neutropenia. In the oncology setting, several of these patients may be treated on an ambulant basis (outside the hospital) [1,2], whereas all patients classified as “high-risk” by MASCC (MASCC < 15) or meeting clinical severity criteria should initially be admitted to the hospital for empirical antibiotic therapy if they are not already hospitalized. Carefully selected “low-risk” patients (e.g., MASCC score > 21) may be candidates for empirical antibiotic therapy on an outpatient basis [2]. To our knowledge, such appropriately-structured and score- or index-guided management is not yet available for idiosyncratic febrile neutropenia cases, although each clinician may have personal experience with such management in selected young patients without comorbidity (e.g., anti-thyroid drug-induced agranulocytosis). Concerning home management, only few retrospective and poorly-designed reports have been published in the scientific literature [5,6].

Our work further highlights the “classical” causative drugs: antibiotics (37.4%); antithyroid drugs (17.2%); neuroleptic and anti-epileptic agents (13.1%); non-steroidal anti-inflammatory agents and analgesics (8%); and platelet aggregation inhibitors (8%), especially ticlopidine. Everyday medicines may thus be implicated in severe accidents. It is, thus, mandatory to appropriately inform and train all practitioners, including those practicing in the city. Our present data were in accordance with those published by Shapiro et al. [22] and van der Klauw et al. [23,24]. In our study, antibiotics were at the top of offending drug classes. It should, however, be noted that in clinical practice, it may often prove challenging to formally attribute neutropenia to a specific drug rather than the infection for which the drug was initiated [5,25]. In our study, the reported cases all fully met the required criteria, as defined in the scientific literature. 

Our study does not enable any conclusion to be drawn about the usefulness of blood cell count monitoring for certain medicines at risk (e.g., antithyroid drugs, ticlopidine, and clozapine), as proposed elsewhere [5,6]. Although patients experiencing idiosyncratic agranulocytosis may initially be asymptomatic or suffer from isolated fever, which was the case in our patients, neutropenia often reflects the onset of severe sepsis depending on its severity and depth [5,6]. Thus, the usefulness of surveillance may be questioned, especially for drugs with a low incidence of idiosyncratic neutropenia.

Our study exhibited several limitations. Chiefly, the data originated from a population covering a period of over 30 years. There may thus be heterogeneity as to the causative drug and possibly the management process. Moreover, all patients were referrals. On the other hand, our study exhibited several strengths, in that it was the first to investigate febrile neutropenia in the setting of established and documented idiosyncratic non-chemotherapy-induced neutropenia. In addition, this study was conducted in a single center, with experienced physicians well- accustomed to managing neutropenia and agranulocytosis. Therefore, it appears difficult to generalize any results and conclusions, and caution is required when extrapolating the findings outside this single center. The small sample size, with only 76 patients included, limited the study’s power to detect any meaningful statistical and clinical patterns. The statistical analyses were thus mostly exploratory without adjusting for any potential confounding factors, and the results could be potentially biased by confounding factors. A larger-scale study with data originating from multiple centers is necessary to confirm our findings, in addition to a sophisticated statistical analysis plan. 

## 5. Conclusions

In conclusion, despite major advances in its prevention and treatment, febrile neutropenia (absolute neutrophil count <0.5 × 10(9)/L) remains a most concerning drug-induced complication, even outside the oncology and myelosuppressive chemotherapy settings. Idiosyncratic febrile neutropenia related to non-chemotherapy drugs is typically serious, with about 40% of patients exhibiting severe sepsis, like pneumonia, septicemia and septic shock, along with a mortality rate of about 10%. As in chemotherapy-related febrile neutropenia, modern management procedures, such as immediate broad-spectrum antibiotherapy and HGF, may reduce infection-related mortality. 

All practitioners should be informed and trained about this potential side-effect, even in the event of “daily medication” exposure. Particular attention should be paid to the following drugs: antibiotics (β-lactams and cotrimoxazole), antithyroid drugs (carbimazole), neuroleptic (clozapine), non-steroidal anti-inflammatory agents and analgesics (noramydopyrine), and platelet aggregation inhibitors (ticlopidine).

## Figures and Tables

**Table 1:** Criteria for inclusion in and exclusion from the study (adapted from [6]).

Inclusion Criteria
Patients Had to Fulfill the Following Conditions *:
- Neutrophil count < 0.5 × 10(9)/L
- Presence of fever, clinical infection, and/or signs of septic shock (chills, sweating, collapse, and confusion)
- Fulfilled standardized criteria by Benichou et al.: agranulocytosis onset within 7 days of treatment in the event of previous intake of the same drug, no clinical features, and >1.5 × 10(9)/L neutrophils in blood cell count 1 month after drug interruption [9]
**Exclusion Criteria:**
- History of congenital neutropenia or immune neutropenia
- No recent viral infection **
- Recent chemotherapy, radiotherapy, and/or immunotherapy
- Existence or development of underlying hematological disease

* For enrolment, patients had to be hospitalized (conventional hospitalization); ** All patients had negative serological tests (IgM) for human immunodeficiency virus, hepatitis B and C virus, Epstein-Barr virus, cytomegalovirus, and parvovirus B19.

**Table 2:** Drugs incriminated in febrile neutropenia related to non-chemotherapy drugs.

Drug Class	Drug
Antibiotics (*n* = 37) *	amoxicillin ± clavulanic acid (*n* = 9), cotrimoxazole (*n* = 6), piperacillin (*n* = 5), ceftriaxone (*n* = 3), teicoplanine (*n* = 3), cefotaxime (*n* = 2), vancomycine (*n* = 2), pristinamycine (*n* = 2), imipenem (*n* = 1), levofloxacin (*n* = 1), ceftazidine (*n* = 1), dalacine (*n* = 1), and ofloxacin (*n* = 1)
Antithyroid drugs (*n* = 17)	carbimazole (*n* = 15), benzylthiouracil (*n* = 1), and thiamazole (*n* = 1)
Neuroleptics and anticonvulsants (*n* = 13) *	cyamemazine (*n* = 3), clozapine (*n* = 2), indalpine (*n* = 2), tiapride (*n* = 2), carbamazepine (*n* = 1), valpromide (*n* = 1), meprobamate (*n* = 1), and valproic acid (*n* = 1)
Platelet aggregation inhibitors (*n* = 8)	ticlopidine (*n* = 5), acid acetylsalicylic (*n* = 3) **
Non-steroidal anti-inflammatory agents and analgesics (*n* = 8) *	ibuprofen (*n* = 4), noramydopyrine (*n* = 3), and tenoxicam (*n* = 1)
Other molecules (*n* = 16) *	valganciclovir (*n* = 2), deferiprone (*n* = 2), salazopyrine (*n* = 1), captopril (*n* = 1), oezomeprazole (*n* = 1), omeprazole (*n* = 1), fluindione (*n* = 1), venlafaxine (*n* = 1), fluoxetine (*n* = 1), mepronizine (*n* = 1), mirtazapine (*n* = 1), clorazepate (*n* = 1), ganciclovir (*n* = 1), and acyclovir (*n* = 1)

* In 12 cases, more than two drugs were suspected to be responsible for IDIA. ** Dose < 300 mg/day.

**Table 3:** Clinical manifestations upon hospitalization for the 76 patients.

Clinical Manifestations	*n* (%)
Isolated fever (unknown origin)	23 (30%)
Sore throat, acute tonsillitis, and maxillary infection	14 (18.4%)
Documented pneumonia	14 (18.4%)
Septicemia	11 (14.5%)
Septic shock	5 (6.5%)
Deep abdominal or pelvic abscess	3 (39%)
Cutaneous infection	2 (2.6%)
Meningites	2 (2.6%)
Cholecystitis	1 (1.3%)
Infectious arthritis or osteonecrosis	1 (1.3%)

**Table 4:** Microbial infections documented in 21 patients (data available for 66 patients).

Microbes	*n* (%)
**Gram-positive cocci:**	12 (18.2%)
*Staphylococcus aureus*	5
Other *Staphylococcus* species	2
*Streptococcus pneumoniae*	4
*Corynebacterium* sp.	1
**Gram-negative bacilli:**	8 (12.1%)
*Escherichia coli*	3
*Pseudomonas aeruginosa* and *Stenotrophomonas maltophilia*	2
*Enterobacter aerogenes* and *E. cloacae*	1
Other Gram-negative bacilli (like *Serratia marsescens*, *Bacteroïdes fragilis*, *Morganella morganii*, *Proteus* sp., and *Prevotella* sp.)	2 *
**Other rarer microorganisms identified**	2 (3%)
*Aspergillus fumigatus*	1
*Mucormycosis* sp.	1
**Sterile multiple bacterial samples**	45 (68.2%)

* evidence of multiple bacteria in various samples.

**Table 5:** Death causes of patients with non-chemotherapy drug-induced agranulocytosis.

Sex	Age	Clinical Picture at Diagnosis	Absolute Number of Neutrophils at Diagnosis (× 10(9)/L)	Therapeutic Management	Cause of Death
F	17	Septicemia (*Escherichia coli*)	0.42	Immediate broad-spectrum IV antibiotics + GCSF	Septic shock
M	47	Pneumonia	0	Immediate broad-spectrum IV antibiotics + amphotericin + GCSF	Septic shock
F	71	Pelvic abscess	0	Immediate broad-spectrum IV antibiotics + GCSF	Septic shock
F	73	Abdominal abscess	0.01	Immediate broad-spectrum IV antibiotics + GCSF	Septic shock
M	81	Septicaemia (*Staphylococcus aureus* and *Pseudomonas aeruginosa*)	0	Immediate broad-spectrum IV antibiotics	Septicemia and worsening of associated comorbidities
F	87	Septicemia (*Staphylococcus aureus*)	0.24	Immediate broad-spectrum IV antibiotics	Cerebral hematoma
F	84	Isolated fever	0.08	Immediate broad-spectrum IV antibiotics	Worsening of associated comorbidities
M	72	Septic shock	0.5	Immediate broad-spectrum IV antibiotics + GCSF	Septic shock

**Table 6:** Protocol for febrile neutropenia management in non-cancer patients.

Immediate arrest of any suspected drugs
Immediate hospitalization
Initial assessment of circulatory and respiratory function, with vigorous resuscitation where necessary, followed by careful search for potential infection source
Systematic multiple microbiological samples (blood, urine, stool, and throat)
Immediate broad-spectrum antibacterial therapy (<1 h after admission) with first-line piperacilline (12 g/day) or cefotaxime (3 g/day), in association with netromycine (5 mg/kg/day) or amikacine (15 mg/kg/day) in sepsis cases, except for β-lactam allergy or β-lactam-induced agranulocytosis. In a second step, the antimicrobial therapy is to be adapted according to microbes and antibiogram results, using mainly glycopeptide antibiotics and penems, in association with amphotericin or voriconazole in fungal infection cases.
Hematopoietic growth factor (GCSF: 300 μg/day)
Daily monitoring of clinical presentation and blood count
Data to be registered in the French national database of drug side-effects
